# Fatal thyrocardiac event

**DOI:** 10.4103/0019-5049.65374

**Published:** 2010

**Authors:** Samit Kumar Khutia, Bhaskar Roy, Mohan Chandra Mandal, Sabyasachi Das

**Affiliations:** Department of Anaesthesiology, NBMC, Sushrutanagar – 734 012, Darjeeling, West Bengal, India

**Keywords:** Atrial fibrillation, anticoagulant, airway management, hyperthyroidism, ischaemic stroke

## Abstract

Atrial fibrillation occurs frequently (2–20%) in chronic hyperthyroidism patients. Poorly treated thyrotoxic patients may present with a life-threatening cerebrovascular accident giving little scope to revert the situation. At times, it is difficult to make a patient euthyroid with conventional management. The definitive treatment of choice is ^131^I, radioiodine. An adjusted dose of an oral anticoagulant is highly efficacious for the prevention of all types of strokes. Timely intervention by a skilled airway manager with right instruments is the key to success in airway management. A 50-year-old thyrotoxic, semiconscious male presented with a sudden onset of haemiplegia. He had chronic AF, a huge thyroid swelling with gross tracheal deviation and dilated cardiomyopathy. A CT scan showed infarction in the left middle cerebral artery territory. After initial improvement with conservative management, patient’s condition deteriorated in the next 48 h. Repeat CT scan showed increase in the infarct size with haemorrhage and midline shift. Finally, he died despite all resuscitative measures.

## INTRODUCTION

Thyroid disorders are the most common endocrine disease in India.[1] Due to close embryological proximity between thyroid gland and heart,[2] patients with long-standing hyperthyroidism predominantly present with different cardiovascular manifestations importantly atrial fibrillation (AF) and heart failure. Prevalence of AF and other arrhythmias in hyperthyroidism is 2–20%, which is again more common with advancing age.[2] Restoring a thyrotoxic patient to an euthyroid state is the primary management of thyrotoxic AF. But inadequate treatment of the thyrotoxicosis leads to chronic AF, which is associated with an increased risk of ischaemic stroke. Large thyroid mass in noneuthyroid patients poses a great challenge to both anaesthesiologists and airway managers.

## CASE REPORT

**Figure 1:**
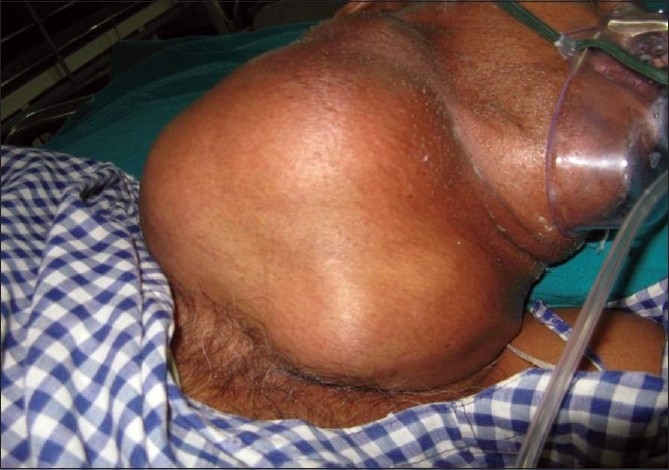
Patient with a huge multinodular thyroid swelling which progressively increased to the present size over the last 20 years

A 50-year-old male homeopath was brought to emergency in a semiconscious state having Glasgow coma scale (GCS) E_2_V_1_M_5_ with right-sided haemiplegia, irregularly irregular pulse and a huge thyroid swelling [Figure 1].

He had a sudden onset of intense throbbing headache since last 6 h with slowly evolving right-sided hemiparesis. He had thyrotoxicosis for last 10 years but received treatment with carbimazole, metoprolol, digoxin, ramipril and aspirin for the last 1 year. Echocardiography showed dilated cardiomyopathy (DCM) with a left ventricular ejection fraction (LVEF) of 40%.

**Figure 2:**
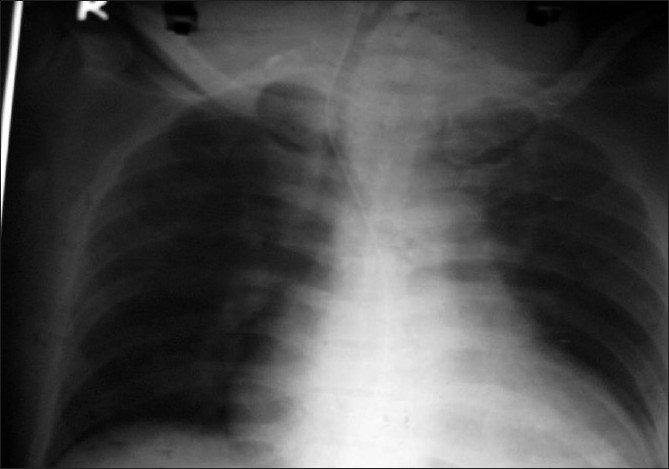
Right-sided deviated trachea with an increased cardiothoracic ratio

**Figure 3:**
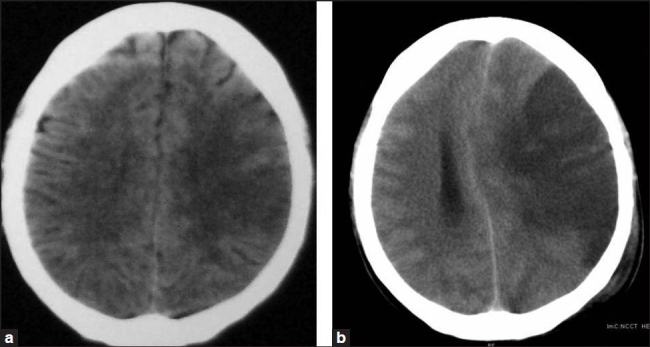
Computed tomography of brain (plain) showed (a) left MCA territory infarction having no midline shift (on admission); (b) large haemorrhagic infarction in the left MCA territory with a gross midline shift after 36 h

On examination he was restless, aphasic, had a heart rate 140/min, irregularly irregular and blood pressure 140/90 mmHg. Pupils were bilaterally mid-dilated, sluggishly reacting to light. Plantar response showed extension on right side and withdrawal on left side. His chest was clear with good bilateral air entry. Peripheral oxygen saturation (SpO_2_) was 99% with O_2_ and oropharyngeal airway. The previous record suggested the absence of retrosternal goitre. Twelve-lead ECG revealed AF. Chest X-ray (CXR) showed gross deviation of trachea to the right side with an increased cardiothoracic ratio [Figure 2]. CT brain (plain) revealed ischaemic infarction in the left middle cerebral artery (MCA) territory [Figure 3a].

Thyroid profile showed TSH 0.037 *μ*U/ml, T_3_ 1.75 nmol/dl and T_4_ 18.68 *μ*g/dl. Other relevant investigations were within normal limits. Patient’s general condition did not permit carotid angiography to establish cardioembolic stroke. A working diagnosis of the cardioembolic stroke was made.

After topical anaesthesia of the airway with nebulised lignocaine (4%) and preoxygenation, rigid laryngoscope-aided fibreoptic orotracheal intubation was done. Intravenous lignocaine (2%) was administered to reduce the stress response. During intubation, the assistant lifted the neck mass anteriorly to facilitate intubation. The nasogastric tube was introduced under direct vision. The patient was put on a ventilator to reduce the work of breathing.

Conservative management started with frusemide, dexamethasone, antibiotics, H_2_-blocker, metoprolol, digoxin and atorvastatin. Tablet carbimazole was given through the nasogastric tube at a dose of 10 mg twice daily. Low-molecular-weight heparin was added after CT scan reporting. General care and necessary monitoring for an unconscious patient was done. The patient’s condition improved after 12 h of admission (GCS E_3_V_T_M_6_) with the heart rate reduced to 98/min. But over the next 36 h, the patient’s condition gradually deteriorated (GCS E_1_V_T_M_4_). Repeat CT revealed large haemorrhagic infarction in the left MCA territory with the gross midline shift [Figure 3b]. Neuromedicine and neurosurgical consultation concluded that surgical decompression would not help because of whole left hemisphere involvement.

After 60 h of admission, the patient became fully unconscious, developed pulmonary oedema and became haemodynamically unstable (heart rate: 150–176/min, BP: 90/60 mmHg and SpO_2_<60%). Vasopressors and other supportive measures were given. Eventually, the patient had a cardiac arrest and died.

## DISCUSSION

AF is the most common arrhythmia with thyrotoxicosis[3] as atria are rich in β-adrenergic receptors.[4] It is well established that AF carries an increased risk of acute ischaemic stroke. Most of the ischaemic strokes with AF, especially with advancing age, are cardioembolic in origin,[5] but 25% are due to co-morbid states, e.g. coronary artery disease (CAD), dilated cardiomyopathy (DCM), hypertension, non-insulin-dependent diabetes mellitus and chronic heart failure.[6] Danish National Registry showed that, among 40628 hyperthyroid patients, although 8.3% developed AF, male gender, ischaemic or valvular heart disease or congestive heart failure were associated with the highest risk rates.[3] Besides, history of alcohol intake, history of previous stroke or transient ischemic attack, smoking, prior myocardial infarction and obesity are potential predictors for AF. Patients with any of these risk factors have an annual stroke risk of at least 4%, if left untreated. The Framingham study[7] showed a 5-fold increase in stroke incidence with chronic AF, whereas AF with rheumatic heart disease (RHD) had a 17-fold increase, compared with controls. The changes in myocardial contractility, left ventricular diastolic performance, systemic vascular resistance and pulmonary vascular resistance, associated with these co-morbid conditions, are responsible for such severe cardiac morbidity in hyperthyroidism.

Early diagnosis and restoring euthyroid state revert AF to sinus rhythm and primarily relate to prognosis.[3] The first-line therapy of AF in the setting of hyperthyroidism includes β-adrenergic blockade[3] which slows the heart rate without altering diastolic performance. At times, it is not possible to achieve euthyroid state with conventional antithyroid drugs. The definitive treatment of choice is ^131^I, radioiodine.[8] This is safe and effective especially when used in conjunction with β-adrenergic blockade.

Anticoagulation of patients with hyperthyroidism and AF is controversial. Though there is an increased risk of oral anticoagulant (OAC)-associated haemorrhage in advanced age,[5] the benefit of OAC with regard to decreased stroke and cardiovascular outcome risk should make OAC the preventive treatment of choice in patients with atrial fibrillation in all age groups (even in the older patients). The target intensity of anticoagulation involves a balance between the prevention of ischaemic stroke and avoidance of haemorrhagic complications which can be achieved with an international normalised ratio (INR) of 2–3.[9] Meta-analysis showed that adjusted-dose OAC is highly efficacious for the prevention of all strokes (both ischaemic and haemorrhagic).[10] Warfarin is far superior to aspirin for preventing cardioembolic strokes, whereas aspirin has its major effect on noncardioembolic events.[6] In addition, antiplatelet therapy – the primary alternative to OAC for patients with atrial fibrillation – becomes significantly less beneficial for stroke prevention as the patient ages.

Patients with large thyroid glands are potentially difficult cases for airway management. If time permits, awake fibreoptic bronchoscope (FOB)-guided intubation is advantageous in tracheal deviation or compression with or without retrosternal goitre. Though nasal FOB intubation is easier, it is relatively contraindicated in patients on OAC. Oral FOB intubation is somewhat more difficult than nasal intubation. But when oral FOB is aided by direct laryngoscopy, it helps in exposing the supraglottic area and suctioning of secretions, allowing smooth passage of FOB beneath the epiglottis and moreover, it saves time.[11]

## CONCLUSION

Though most of the patients recover completely from the abnormal cardiac functions after the treatment of the hyperthyroidism only, some patients are resistant to conventional management. A thyrocardiac event could be the primary presentation. Anaesthesiologists should be aware of this complication which may arise in the perioperative period too. Airway management in these patients is very challenging.
